# Lorlatinib and capmatinib in a *ROS1*-rearranged NSCLC with *MET*-driven resistance: tumor response and evolution

**DOI:** 10.1038/s41698-023-00464-y

**Published:** 2023-11-03

**Authors:** Jaime L. Schneider, Khvaramze Shaverdashvili, Mari Mino-Kenudson, Subba R. Digumarthy, Andrew Do, Audrey Liu, Justin F. Gainor, Jochen K. Lennerz, Timothy F. Burns, Jessica J. Lin

**Affiliations:** 1 Massachusetts General Hospital Cancer Center and Department of Medicine, Boston, MA 02114 USA; 2 Harvard Medical School, Boston, MA 02115 USA; 3 University of Pittsburgh Medical Center Hillman Cancer Center, Pittsburgh, PA 15219 USA; 4 Department of Medicine, Division of Hematology Oncology, University of Pittsburgh School of Medicine, Pittsburgh, PA 15219 USA; 5 Center for Integrated Diagnostics, Department of Pathology, Massachusetts General Hospital, Boston, MA 02114 USA

**Keywords:** Non-small-cell lung cancer, Cancer genomics

## Abstract

Acquired drug resistance remains a major problem across oncogene-addicted cancers. Elucidation of mechanisms of resistance can inform rational treatment strategies for patients relapsing on targeted therapies while offering insights into tumor evolution. Here, we report acquired *MET* amplification as a resistance driver in a *ROS1*-rearranged lung adenocarcinoma after sequential treatment with ROS1 inhibitors. Subsequent combination therapy with lorlatinib plus capmatinib, a MET-selective inhibitor, induced intracranial and extracranial tumor response. At relapse, sequencing of the resistant tumor revealed a *MET* D1246N mutation and loss of *MET* amplification. We performed integrated molecular analyses of serial tumor and plasma samples, unveiling dynamic alterations in the ROS1 fusion driver and MET bypass axis at genomic and protein levels and the emergence of polyclonal resistance. This case illustrates the complexity of longitudinal tumor evolution with sequential targeted therapies, highlighting challenges embedded in the current precision oncology paradigm and the importance of developing approaches that prevent resistance.

## Introduction

Chromosomal rearrangements involving the ROS proto-oncogene 1 (*ROS1*) gene are potent oncogenic drivers identified across multiple tumor types including in 1–2% of non-small cell lung cancer (NSCLC)^1,2^. *ROS1* rearrangements result in the expression of the ROS1 fusion oncoprotein, constitutive ROS1 kinase activity, and aberrant downstream signaling activation, ultimately leading to dysregulated tumor survival and proliferation^3^. Patients with metastatic *ROS1-*rearranged lung cancers typically derive substantial benefit from ROS1-targeted tyrosine kinase inhibitors (TKIs) such as crizotinib and entrectinib, often with marked initial tumor responses^4,5^. However, the durability of response is inevitably limited in most patients due to acquired drug resistance. Prior studies have elucidated mechanisms of on-target resistance mediated by a spectrum of *ROS1* kinase domain mutations (e.g., *ROS1* G2032R)^6–9^, motivating the clinical development of next-generation ROS1 inhibitors^10–13^. In contrast, insights into mechanisms of off-target resistance have remained limited.

Amplification of the mesenchymal-epithelial transition (*MET*) gene—which encodes a receptor tyrosine kinase involved in promoting mitosis, motility, and survival^14^—represents one potentially actionable mechanism of off-target resistance to ROS1 inhibitors that has been identified using *ROS1*-rearranged cell lines and biopsies from patients relapsing on therapy (including after entrectinib and lorlatinib)^9,15,16^. In *EGFR*-mutated lung cancers harboring *MET* amplification-mediated resistance to EGFR TKIs (which occurs in 10–15% of patients relapsing on first-line third-generation EGFR inhibitors)^17,18^, various combinations of EGFR and MET TKIs have demonstrated clinical activity and tolerability in phase I/II clinical trials^19,20^, attesting to the actionability on this resistance driver. Similarly, combinations with MET inhibitors have induced tumor responses in *ALK-* and *RET*-rearranged lung cancers with MET-driven resistance to TKIs targeting the original oncogene drivers^21,22^. In *ROS1*-rearranged disease with MET-mediated resistance, the optimal clinical strategy for co-targeting ROS1 and MET is unknown. One case report described the efficacy of crizotinib monotherapy (a multitargeted ROS1/MET/ALK TKI) in a patient relapsing on entrectinib with *MET* polysomy^23^. Crizotinib is a first-generation multikinase inhibitor with limited selectivity, suboptimal coverage of on-target resistance mutations that can emerge on therapy, and poor blood-brain barrier penetrance^24^, all of which can limit clinical benefit. Therefore, a rationale exists for exploring combination strategies that exploit potent, CNS-penetrant next-generation ROS1 TKIs and selective MET TKIs such as capmatinib or tepotinib.

Of note, across treatment-naïve and -resistant cancers, upregulation of MET signaling occurs through an array of molecular events ranging from protein overexpression to genomic alterations, including amplification, mutations (e.g., exon 14 skipping), or chromosomal rearrangements^25^. These molecular alterations have distinct functional consequences and clinical implications^26^. The MET-selective TKIs capmatinib and tepotinib are FDA-approved for the treatment of advanced lung cancers harboring *MET* exon 14 skipping^27,28^. However, both TKIs have additionally demonstrated efficacy in *MET*-amplified NSCLC^28,29^. By comparison, MET overexpression is not currently considered an actionable biomarker from the perspective of TKI therapy, although this is anticipated to evolve with the advent of non-TKI therapeutic agents targeting MET, such as antibody–drug conjugates (ADCs)^30,31^.

Here, we report a case of *ROS1*-rearranged lung cancer overexpressing MET protein at baseline, which then acquired genomic amplification of *MET* as an off-target mechanism of resistance to ROS1 inhibitors. To our knowledge, this is the first documented case of extracranial and intracranial disease response to a ROS1/MET co-inhibition strategy using lorlatinib plus capmatinib in *MET*-amplified, *ROS1*-rearranged NSCLC. The subsequent emergence of an on-target *MET* resistance mutation and loss of *MET* amplification highlights the temporal evolution of multi-faceted MET signaling axis activation and complex overall molecular landscape longitudinally as tumor cells acquire resistance to sequential targeted therapies.

## Results

### Case

**Fig. 1:**
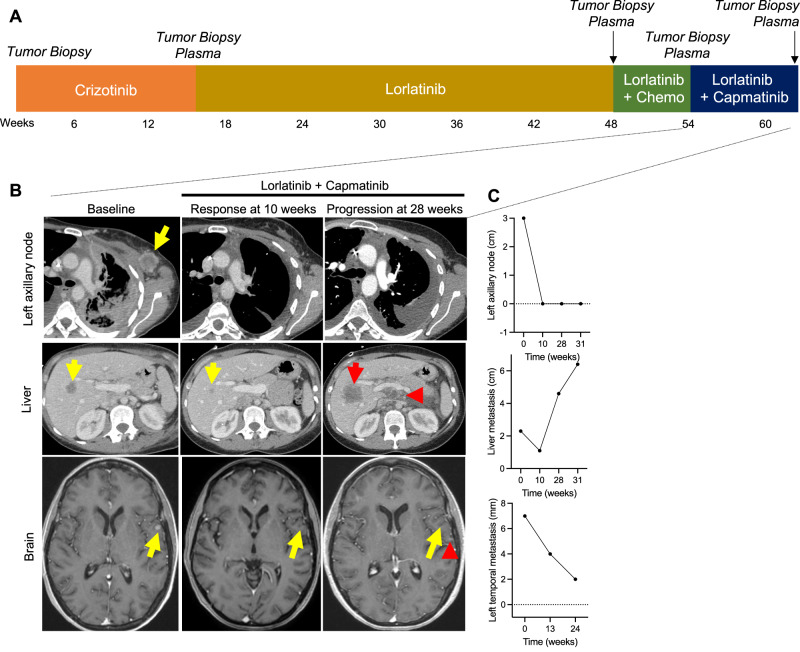
Treatment history and evolution of tumor burden on lorlatinib and capmatinib. **A** Timeline summarizing the treatment course and tumor and plasma sampling. Chemo: chemotherapy. **B** Representative computed tomography (CT) and brain magnetic resonance imaging (MRI) images demonstrating response to, and progression on, lorlatinib plus capmatinib therapy at the indicated time points, including the response of left axillary lymph node (yellow arrow), chest wall disease (yellow arrow), pleural thickening (yellow arrowhead), and liver metastasis (yellow arrow) to lorlatinib plus capmatinib dual therapy at 10 weeks; increasing liver metastasis (red arrow), new abdominal lymphadenopathy (red arrowhead), and new left temporal brain metastasis (red arrowhead). **C** Measurements on indicated metastatic lesions over the treatment course. Time 0 = baseline scan prior to initiation of lorlatinib plus capmatinib.

A 34-year-old woman with no smoking history presented with persistent dry cough and was found to have a 2.2 cm FDG-avid left lingular lung nodule on chest computed tomography (CT) and positron emission tomography (PET) imaging without evidence of metastatic disease. A biopsy of the lung nodule demonstrated primary lung adenocarcinoma. She underwent left upper lobe segmentectomy but was noted intraoperatively to have pleural and pericardial nodules. Cytology evaluation demonstrated malignant pleural effusion, and pathologic staging was pT2 pNx pM1a. Genotyping via next-generation sequencing (NGS) and ROS1 fluorescence in situ hybridization (FISH) on the resected lung tumor sample revealed a *SLC34A2-ROS1* gene rearrangement and *CDKN2A* H83Y and *TP53* V173fs mutations detected on NGS (Supplementary Table 1). ROS1 protein expression was confirmed by immunohistochemistry (IHC). She was initiated on first-line crizotinib, 250 mg twice daily, with the initial response (Fig. 1A). After 14 months, imaging showed evidence of progression with new hepatic metastases. IR-guided liver biopsy confirmed adenocarcinoma with *ROS1* rearrangement by NGS testing and FISH (Supplementary Table 1). The patient was started on second-line lorlatinib, dose-reduced to 75 mg for neurocognitive adverse events (AEs). After 30 months, scans showed intrathoracic and abdominal progression with peritoneal carcinomatosis. Thoracentesis of the left pleural effusion revealed recurrent adenocarcinoma. NGS testing again demonstrated the *SLC34A2-ROS1* rearrangement, *TP53* V173fs, *ATM* H2075R (VUS), *PMS2* E518K (VUS), *ERCC2* N92S (VUS) (Supplementary Table 1). Platinum-doublet chemotherapy (carboplatin and pemetrexed) was initiated with continued lorlatinib 50 mg daily.

**Fig. 2:**
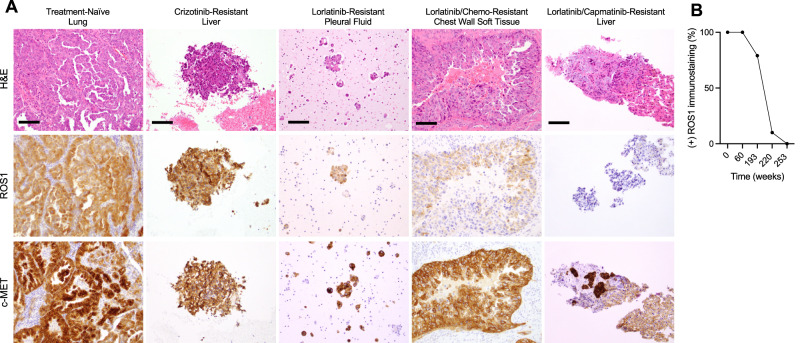
ROS1 and MET protein expression during the treatment course. **A** Representative immunohistochemistry (IHC) images demonstrating the H&E, ROS1, and c-MET immunostaining at indicated time points. Scale bar: 100 µm. **B** Percentage of tumor cells immunostaining positive for ROS1 at same time points as shown in (**A**).

**Fig. 3:**
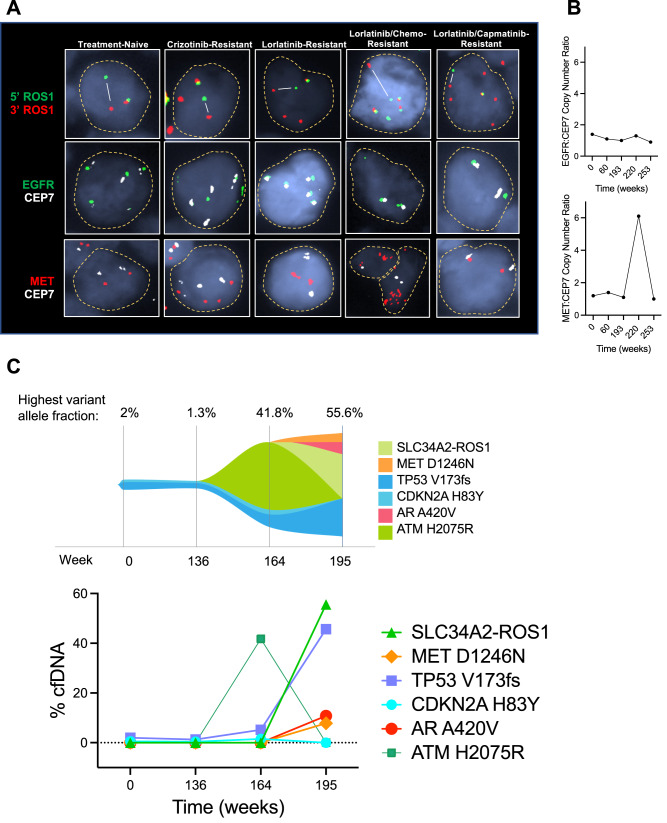
Longitudinal evolution of genomic alterations including *MET* amplification and resistance mutation. **A** Representative fluorescence in situ hybridization (FISH) images for *ROS1* rearrangement (top), *EGFR* copy number relative to *CEP7*, and *MET* copy number relative to *CEP7* (bottom). Split signals (white lines) denote *ROS1* rearrangement. **B** The EGFR-to-CEP7 ratio (i.e., the ratio of the green signals to the white signals) and MET-to-CEP7 ratio (i.e., the ratio of the red signals to the white signals) are shown at the indicated time points throughout the disease course. **C** Guardant360 cell-free DNA (cfDNA) response map showing dynamic changes in variant allele fractions of gene alterations. The evolution of variant alleles over time, represented as % cfDNA, is depicted (bottom). Timepoint 0 = plasma liquid biopsy prior to starting lorlatinib treatment.

After an initial response to chemotherapy plus lorlatinib, the patient presented with intracranial and extracranial progressive disease after 5.5 months (Fig. 1), including new multifocal brain parenchymal lesions, worsening bilateral pleural effusions, enlarging left axillary lymphadenopathy, and a left-sided chest wall soft tissue mass biopsy-proven to be metastatic lung adenocarcinoma without evidence of histologic transformation. DNA-based NGS testing on the chest wall mass revealed the known *SLC34A2-ROS1* rearrangement and *CDKN2A* H83Y and *TP53 V173fs* mutations (Supplementary Table 1). *ROS1* FISH assay confirmed retention of the genomic *ROS1* rearrangement (Fig. 3A). However, IHC analysis showed a new loss of ROS1 protein expression (Fig. 2A, B). Additionally, NGS testing revealed a *de novo MET* amplification (7q31 gain) which was confirmed by *MET* FISH testing with a MET:CEP7 ratio of 6.1 (Fig. 3A, B). Of note, MET IHC confirmed expression of MET at this time point (*H*-score 130) (Fig. 2A). However, upon retrospective testing, MET overexpression was detected in all prior biopsies, including in the treatment-naïve lung tumor at initial diagnosis and in the crizotinib- and lorlatinib-resistant tumor specimens (Fig. 2A; *H*-scores 300 and 300, respectively). In contrast, genomic *MET* amplification (as tested using *MET* FISH) was absent in all previously biopsied tumors.

Given the evidence of acquired *MET* amplification, and in view of active disease in the central nervous system (CNS), combination therapy of lorlatinib (dose reduced to 50 mg daily) and capmatinib (400 mg twice daily), an ATP-competitive, potent, and CNS-penetrant type Ib MET TKI^28^, was pursued. She experienced rapid clinical improvement with palpable shrinkage of left axillary fullness and improvement of fatigue and shortness of breath within 2 weeks. Repeat scans performed 10 weeks after initiation of combination therapy demonstrated partial response per RECIST v1.1 criteria with a 69% reduction in the target tumor lesions (left axillary lymph node, hepatic metastasis) as well as shrinkage of non-target lesions, including the left pleural thickening and chest wall soft tissue disease (Fig. 1B, C, Supplemental Table 1). Furthermore, a brain MRI demonstrated shrinkage of numerous parenchymal CNS metastases, including a 43% reduction in the dominant left temporal lobe lesion, consistent with intracranial partial response per modified RECIST v1.1. She continued the combination for a total of 32 weeks until disease progression, with treatment-emergent AE of grade 1 lower extremity edema and nausea attributed to capmatinib (predominantly) and lorlatinib and grade 2 hyperlipidemia attributed to lorlatinib. No dose interruptions or dose reductions due to treatment-emergent AEs were required.

Subsequently, due to progression, including increased hepatic metastases, portocaval lymphadenopathy, malignant ascites, and recurrence of brain metastases (Fig. 1B, C), the combination regimen was discontinued. Metastatic disease in the left axillary node, left pleura, and left chest wall were continuing to respond, indicating heterogeneity across tumor sites. Repeat liver biopsy showed retained adenocarcinoma histology and persistent loss of ROS1 expression by IHC. NGS testing revealed the original *ROS1* rearrangement, and a *de novo MET* D1246N mutation (Supplementary Table 1), known to confer resistance to type I MET TKIs, including crizotinib, capmatinib, and tepotinib^32–34^. Of note, the previously detected *MET* amplification was no longer identified on *MET* FISH testing (MET:CEP7 copy number ratio, 1.1). Liquid biopsy with Guardant360 cell-free DNA (cfDNA) assay demonstrated a *SLC34A2-ROS1* rearrangement [variant allele fraction (VAF) 55.6%] and heterogeneous genomic alterations, including *MET* D1246N (VAF 7.8%) and *TP53* V173fd (VAF 45.7%), as well as *BRCA2* loss, *EGFR* amplification (medium-level, plasma copy number 3), and *FGFR1* amplification (low-level, plasma copy number 2.4) which had not been identified through tissue NGS or *EGFR* FISH testing (Fig. 3C, Supplementary Table 1). The patient was offered cabozantinib, a multikinase type II inhibitor with ROS1/MET-targeted activity^35^, given its potency against the *MET* D1246N resistance mutation^36^. The patient received three days of cabozantinib before being hospitalized for worsening ascites and ultimately decided against further treatment and was transitioned to comfort-focused care.

## Discussion

This report represents, to the best of our knowledge, the first description of a dramatic—albeit brief—clinical and radiographic tumor response to combined selective inhibition of ROS1 and MET using lorlatinib plus capmatinib in the setting of acquired resistance to sequential ROS1 inhibitor therapy (crizotinib followed by lorlatinib) due to *MET* amplification. In this case, the combination of lorlatinib plus capmatinib was chosen and preferred over alternative options (crizotinib monotherapy or lorlatinib plus crizotinib) in order to maximize CNS penetration and activity in the context of actively progressing brain metastases. Indeed, the patient experienced both intracranial and extracranial responses to therapy.

The activity of lorlatinib plus capmatinib in this patient with *ROS1*-rearranged cancer confirms *MET* amplification as the driver of resistance that is, importantly, actionable. Collective evidence continues to buttress *MET* amplification as a shared genomic mechanism of off-target resistance and biomarker for subsequent rational combination strategy across oncogene-addicted lung cancers^19,21,22^, and underscores the clinical value of re-biopsy and comprehensive molecular profiling in therapy-resistant cancers. In addition, here, the combination of half-dose lorlatinib and full-dose capmatinib was tolerated without grade 2 or higher treatment-related AEs or the requirement for further dose modifications. It is impossible to generalize regarding the efficacy or tolerability of lorlatinib plus capmatinib in patients with *MET*-amplified, *ROS1*-rearranged lung cancers on the basis of this single case. However, it will not be pragmatically feasible to perform phase Ib/II clinical trials of discrete combination regimens for each individual therapy-resistant, rare molecular subset of lung cancer (e.g., *ROS1-*, *RET-*, and *NTRK1-3*-rearranged NSCLC) and for each distinct genomic mechanism of resistance. Therefore, creative basket trial designs in parallel with added knowledge from smaller case series and extrapolation from the available data in relatively larger molecular subsets of NSCLC (such as *EGFR*-mutant disease) will be pivotal to effectively inform clinical practice and advance viable treatment strategies. For instance, the field may benefit from shifting the way basket trials are structured to be centered around mechanisms of resistance rather than each original oncogenic driver.

The brief duration of benefit from the combination regimen herein is noteworthy and deemed to reflect the emergent tumor heterogeneity across disease sites under the selective pressure of sequential targeted therapies—a prominent recurring clinical problem within the evolving treatment paradigm^37^. Indeed, at the time of disease progression on lorlatinib plus capmatinib therapy, a subset of the known metastatic foci of disease were continuing to respond, whereas others had developed resistance—demonstrating clearly discordant inter-metastatic tumor behavior. Furthermore, the serial cfDNA findings were supportive of genotypic polyclonal resistance, with identification in the lorlatinib/capmatinib-resistant sample of new *BRCA2* loss, *EGFR* amplification, and *FGFR1* amplification (all likely subclonal events) on top of the known *ROS1* rearrangement, *TP53* variant, and *MET* D1246N resistance mutation that had been detected in the contemporaneous liver tumor biopsy. These findings highlight the complementary advantage of liquid biopsy in capturing heterogeneous resistance mechanisms that may exist across distinct metastatic sites within a patient and are missed in single-lession tumor biopsies. In addition, the complex evolution of resistance—as exemplified by (1) the stepwise acquisition of lorlatinib-refractory *MET* amplification followed then by capmatinib-refractory *MET* D1246N mutation with loss of *MET* amplification and (2) the emergence of polyclonal resistance-underscores that therapeutic strategies aimed at delaying or preventing resistance are ultimately more likely to be truly transformative than those aimed at overcoming resistance once it has already developed.

We have additionally uncovered multiple layers of MET axis activation in this case, differentially engaged throughout distinct therapy exposures. MET was overexpressed in the treatment-naïve tumor, and this persisted throughout the patient’s disease course. Despite the protein overexpression of MET, in the absence of additional genomic alterations in *MET*, tumor cells were ROS1-dependent, as evidenced by the initial durable responses to ROS1 inhibitors crizotinib (14 months) and then lorlatinib (30 months). The subsequent *MET* amplification event likely served to engage MET signaling as a true bypass pathway, driving the resistance to ROS1-targeted therapy and conferring MET dependency. Thus, this case supports that high-level expression of MET alone without concomitant genomic lesions such as amplification is an insufficient oncogene or resistance driver. Moreover, the case illustrates that distinct mechanisms of MET activation are not functionally equivalent, emphasizing the importance of understanding the differences in various diagnostic methods used (such as differences between FISH, IHC, and NGS testing for *MET*), to enable accurate interpretation and clinical application of the results.

Intriguingly, we found that ROS1 protein expression was ‘lost’ despite retained genomic *ROS1* rearrangement at the time of *MET* amplification-driven ROS1 TKI resistance. The loss of ROS1 expression in the resistant tumor—while concordant with phenotypic lack of sensitivity to ROS1 TKI—raises important questions about the continued molecular changes involving the original oncogenic driver during targeted therapy. Case reports have detailed the loss of fusion or mutant oncoprotein expression in lineage-transformed TKI-resistant lung cancers, including *EGFR*-mutant and *ROS1*-rearranged NSCLC^38,39^. Furthermore, studies in cell culture models have demonstrated that reduction of ROS1 protein expression or phosphorylation can also occur in the context of ROS1 TKI resistance and activation of off-target bypass pathways^15,40^. The genetic and epigenetic mechanisms mediating the downregulation or loss of the original oncoprotein expression remain to be elucidated.

In summary, this report confirms that *MET* amplification is a bona fide driver of ROS1 TKI resistance in *ROS1*-rearranged lung cancer and that a combination of CNS-penetrant and selective MET and ROS1 inhibitors can induce intra- and extracranial responses. Our findings from the integrated clinical and molecular analyses leveraging multiple serial tumor and plasma samples highlight both the successes and salient challenges embedded in the current era of precision oncology, including the stepwise acquisition of distinct resistance mechanisms with sequential targeted therapies, dynamic and multi-faceted changes involving the original oncogenic driver and the bypass signaling axis, and the emergence of polyclonal resistance resulting in therapy-refractory tumor. Efforts for comprehensive molecular testing at the time of acquired drug resistance will continue to be clinically relevant and potentially guide subsequent therapy selection. Yet these efforts will need to be complemented by discovery efforts focused on developing avenues for fundamentally altering the natural course of cancer and circumventing the evolution of resistance upfront.

## Methods

### Participant

The patient was treated with commercial supplies of lorlatinib plus capmatinib (off-label use) after a detailed discussion of the potential benefits and risks of the therapeutic regimen. The patient provided informed consent for the treatment. The patient signed written informed consent under an institutional review board (IRB)-approved protocol for electronic medical record review to extract relevant clinicopathologic data, including treatment history and outcomes, and for all indicated sample collections and analyses. The radiographic responses to therapy were assessed by an independent radiologist (S.R.D.) according to the RECIST version 1.1 criteria for extracranial disease and modified RECIST version 1.1 criteria for intracranial disease. AEs were graded according to the CTCAE version 5.0.

### Materials and molecular testing

Tumor samples were obtained either by surgical resection, core needle biopsy, or thoracentesis. Formalin-fixed paraffin-embedded specimens derived from tumor samples were reviewed by an MGH pathologist (M.M.K.) to confirm the histopathological diagnosis and to ensure adequate tumor cellularity for analysis. All NGS testing was performed on a clinical basis using extracted DNA and amplicon sequencing with the UPMC Oncomine platform in Pittsburgh, PA. *ROS1* rearrangement and *EGFR* and *MET* copy number gains were assessed using FISH. Briefly, 5-micron sections of formalin-fixed paraffin-embedded tumor material were prepared, and an H&E section was reviewed to select regions for hybridization that contain a majority of tumor cells. A break-apart probe set targeting the 5′ upstream region of ROS1 (RP11-359J11) and the 3′ downstream region of ROS1 (RP11-1036C2) was hybridized, as previously described^41^. The number of cells containing break-apart signals or rearrangement was scored out of a total 50-cell count. A ROS1 rearrangement was reported if more than 15% of cells show split signals. For EGFR, we used a dual-color FISH assay using Bacteria Artificial Chromosome probe CTD-211A18 (chromosome 7p EGFR locus) and a copy number control (centromere 7; Abbott-Vysis), and for MET, we used a dual-color FISH assay using Bacterial Artificial Chromosome probe CTB-13N12 (chromosome 7q MET locus) and a copy number control (centromere 7 or CEP7; Abbott-Vysis). Signal quantitation was used to generate an EGFR or MET to centromere 7 ratio. For example, a ratio of MET:CEP7 of 1.8 to 2.2 is considered borderline for amplification. A ratio between 2.2 to 5.0 is considered low-level amplification. A ratio greater than 5.0 or clustered MET signals that are too numerous to count would be considered highly amplified^42,43^. ROS1 and MET IHC were performed with Automated Stainer Leica Bond III (Leica Biosystems, Danvers, MA) using a ROS1 antibody (clone Rabb4, catalog number 32875, dilution 1:200, Cell Signaling Technology, Danvers, MA) and a c-MET antibody (clone SP44, catalog number 790-4430, Roche Diagnostics, Tucson, AZ), respectively. All procedures were performed in a CLIA-compliant environment.

### Ethics

The Dana-Farber/Harvard Cancer Center Institutional Review Board (DFCI #13-416) approved this study.

### Reporting summary

Further information on research design is available in the Nature Research Reporting Summary linked to this article.

### Supplementary information

supp table

Reporting Summary

## Data Availability

All the data generated for this study are available in this article or from the corresponding author upon request.
