# Supplementary material for: Lorlatinib and capmatinib in a ROS1-rearranged NSCLC with MET-driven resistance: tumor response and evolution
Source: NPJ Precis Oncol. 2023 Nov 3;7:116. doi: 10.1038/s41698-023-00464-y (PMC10624912; doi:10.1038/s41698-023-00464-y)
Supplement: Supplementary file 1 — supp table [file 41698_2023_464_MOESM1_ESM.pdf]

SUPPLEMENTAL TABLE 1. Tissue and plasma molecular testing results.

Weeks since diagnosis	Treatment prior to biopsy	Tissue Molecular Testing							Plasma Genotyping			
		Tissue site biopsied	Tissue NGS platform	*ROS1* alterations in tissue	ROS1 protein expression by IHC	*MET* alterations in tissue	MET protein expression by IHC, H-score	Other variants detected by tissue NGS	Plasma NGS platform	Plasma mutations (variant allele fraction)	Plasma CNAs (plasma copy number)	Plasma fusions
**0**	**Treatment-naive**	Lung	NGS Panel for Lung Tumors, University of Pittsburgh, PA	SLC34A2-ROS1<br>No ROS1 mutations	100%	None	260	CDKN2A H83Y<br>TP53 V173fs	N/A	N/A	N/A	N/A
**60**	**Crizotinib**	Liver	UPMC Oncomine, Pittsburgh, PA	SLC34A2-ROS1<br>No ROS1 mutations	100%	None	300	CDKN2A H83Y<br>TP53 V173fs<br>Copy number LOSS:<br>9p region (CDKN2B)<br>12p region (CCND2,CDKN1B)<br>18q region (SMAD4)	Guardant360	CDKN2A (0.5%)<br>TP53 V173fs (2%)	None	None
**193**	**Lorlatinib**	Pleural fluid	UPMC Oncomine, Pittsburgh, PA	SLC34A2-ROS1<br>No ROS1 mutations	70%	None	300	TP53 V173fs<br>ATM H2075R<br>PMS2 E518K<br>ERCC2 N92S	Guardant360	CDKN2A (0.4%)<br>TP53 V173fs (1.3%)	None	None
**220**	**Lorlatinib plus chemotherapy**	Chest wall	UPMC Oncomine, Pittsburgh, PA	SLC34A2-ROS1<br>No ROS1 mutations	10%	MET amplification (7q31 gain)<br>FISH: MET/CEP7 ratio: 6.1	130	CDKN2A H83Y<br>TP53 V173fs<br>ATM H2075R<br>PMS2 E518K<br>ERCC2 N92S	Guardant360	CDKN2A (1.5%)<br>TP53 V173fs (5.2%)<br>ATM H2075R (41.8%)	None	None
**253**	**Lorlatinib plus capmatinib**	Liver	UPMC Oncomine, Pittsburgh, PA	SLC34A2-ROS1<br>No ROS1 mutations	0%	MET D1246N	300	CDKN2A H83Y<br>TP53 V173fs<br>PMS2 E518K<br>ERCC2 N92S	Guardant360	TP53 V173fs (45.7%)<br>AR A420V (10.8%)<br>MET D1246N (7,8%)	BRCA2 loss (1.7)<br>EGFR amplification (3)<br>FGFR1 amplification (2.4)	SLC34A2-ROS1

Abbreviations: NGS, next-generation sequencing; IHC, immunohistochemistry; CNAs, copy number alterations.
